# Economic evaluation of COVID-19 vaccination: A systematic review

**DOI:** 10.7189/jogh.13.06001

**Published:** 2023-01-14

**Authors:** Auliasari Meita Utami, Farida Rendrayani, Qisty Aulia Khoiry, Dita Noviyanti, Auliya A Suwantika, Maarten J Postma, Neily Zakiyah

**Affiliations:** 1 Department of Pharmacology and Clinical Pharmacy, Faculty of Pharmacy, Universitas Padjadjaran, Bandung, Indonesia; 2 Center of Excellence for Pharmaceutical Care Innovation, Universitas Padjadjaran, Bandung, Indonesia; 3 Department of Health Sciences, University of Groningen, University Medical Center Groningen, Groningen, the Netherlands; 4 Department of Economics, Econometrics & Finance, University of Groningen, Faculty of Economics & Business, Groningen, the Netherlands

## Abstract

**Background:**

Safe and effective vaccination is considered to be the most critical strategy to fight coronavirus disease 2019 (COVID-19), leading to individual and herd immunity protection. We aimed to systematically review the economic evaluation of COVID-19 vaccination globally.

**Methods:**

We performed a systematic search to identify relevant studies in two major databases (MEDLINE/PubMed and EBSCO) published until September 8, 2022. After deduplication, two researchers independently screened the study titles and abstracts according to pre-determined inclusion and exclusion criteria. The remaining full-text studies were assessed for eligibility. We assessed their quality of reporting using the Consolidated Health Economic Evaluation Reporting Standards (CHEERS) 2022 checklist and summarized and narratively presented the results.

**Results:**

We identified 25 studies that assessed the economic evaluation of COVID-19 vaccination worldwide by considering several input parameters, including vaccine cost, vaccine efficacy, utility value, and the size of the targeted population. All studies suggested that COVID-19 vaccination was a cost-effective or cost-saving intervention for mitigating coronavirus transmission and its effect in many countries within certain conditions. Most studies reported vaccine efficacy values ranging from 65% to 75%.

**Conclusions:**

Given the favorable cost-effectiveness profile of COVID-19 vaccines and disparities in affordability across countries, considering prioritization has become paramount. This review provides comprehensive insights into the economic evaluation of COVID-19 vaccination that will be useful to policymakers, particularly in highlighting preventive measures and preparedness plans for the next possible pandemic.

The pandemic of coronavirus disease 2019 (COVID-19) caused by severe acute respiratory syndrome coronavirus 2 (SARS-CoV-2) is a significant public health problem that has affected millions of people globally [1,2]. The virus first appeared in a cluster of patients with pneumonia-like symptoms in Wuhan, China, near the end of 2019 [3]. The disease has put public health systems under pressure [3,4] because of its rapid and intense transmission [3,5], while causing immense economic losses due to medical expenditures and decreased productivity. The estimation of global economic costs of COVID-19 are varied, ranging from US$77 billion to US$2.7 trillion [6], with estimated years of life lost (YLLs) as high as 4 072 325 in 30 high-incidence countries in the first year of the pandemic [7]. Preventive control measures have become a priority due to the lack of an effective and clinically proven pharmacological treatment [5,8,9]. They include nonpharmacological interventions such as isolation and quarantine, cleaning and disinfection, proper use of face masks, and physical distancing [8,10]. The most important strategy, however, is safe and effective vaccination, as it helps with achieving better herd immunity faster [3,4,11].

By September 2022, various vaccines have been developed by many countries. According to the World Health Organization (WHO), there were about 369 vaccine candidates in development, with around 40% in clinical trials, and the remaining 60% in pre-clinical development stages [12]. After a series of efficacy and safety assessments almost two years into the pandemic, numerous COVID-19 vaccines have received Emergency Use Listing (EUL) or Emergency Use Authorization (EUA) by regulatory authorities worldwide, and vaccinations have been conducted in many countries [13]. While some COVID-19 vaccines appear safe and effective, providing an adequate number of vaccines is frequently dependent on the countries' resources [14]. Although the WHO has published guidelines for vaccine prioritization, only a few include economic considerations [15].

A recent study that assessed the duration of effectiveness of COVID-19 vaccines found that, although the COVID-19 vaccine’s immediate effectiveness in preventing severe disease symptoms remained high, its effectiveness may decrease in the six months following full vaccination [16]. These findings highlight that further follow-up on COVID-19 vaccination policies is still required. Given the disease’s health and economic burden, providing information on the effectiveness and cost of health interventions is essential for informing decision-makers in optimizing the scarce healthcare resources, especially in countries with limited resources such as in low- and middle-income countries (LMICs). A previous study showed that nonpharmacological interventions, vaccinations, and treatments can be cost-effective interventions to prevent and control COVID-19 [17]. A most recent review also suggested that COVID-19 vaccination was cost-effective and even cost-saving in LMICs [18]. However, studies that comprehensively assessed the cost-effectiveness of COVID-19 vaccination are currently sparse. To address this, we aimed to conduct a systematic review to assess and provide an up-to-date economic evaluation of COVID-19 vaccination globally.

## METHODS

We followed the Preferred Reporting Items for Systematic Reviews and Meta-Analysis (PRISMA) 2020 guidelines in reporting this systematic review. The PRISMA checklists of this study are provided in Table S1 in the **Online Supplementary Document**.

### Search strategy

Three investigators (AMU, FR, and QAK) searched the MEDLINE/PubMed and EBSCO databases up to September 8, 2022 to identify relevant studies on economic evaluations of COVID-19 vaccination. The following keywords were used for the search, combining mesh terms and text words: (“Costs and Cost Analysis”[Mesh] OR “economic evaluation” OR “cost minimization” OR “Cost-Effectiveness Analysis”[Mesh] OR “cost utility” OR “Cost-Benefit Analysis”[Mesh] OR “willingness-to-pay”) AND (“COVID-19”[Mesh] OR “Coronavirus”[Mesh] OR “COVID-19 Vaccines”[Mesh]).

### Study selection

**Figure 1:**
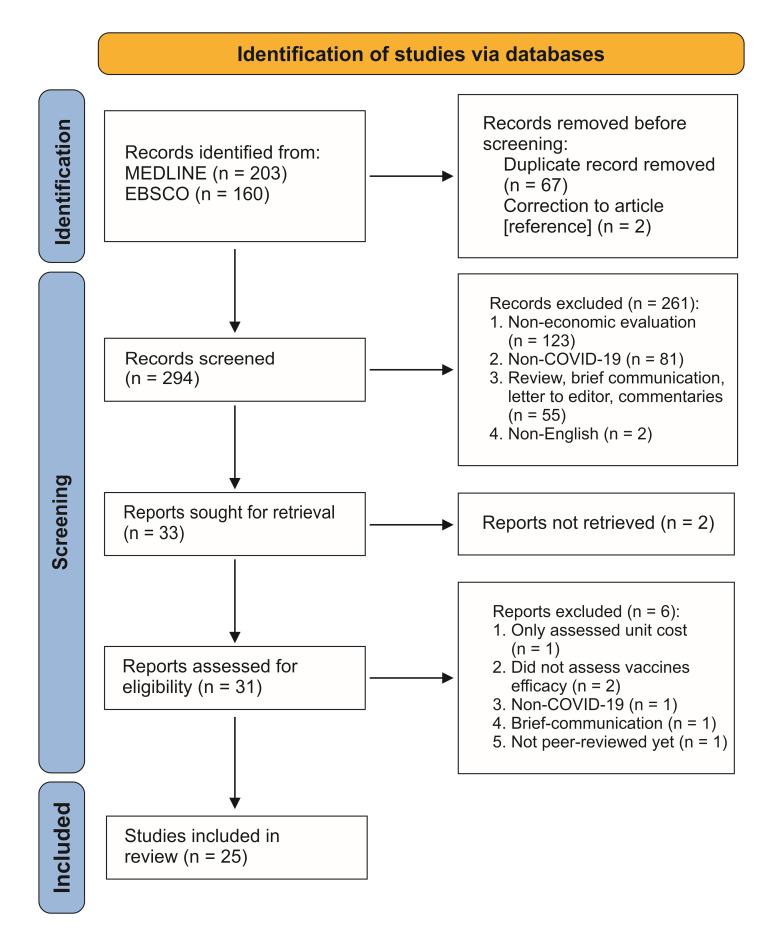
PRISMA flow diagram of the study selection process.

We exported the records into the Mendeley Reference Manager and checked for duplicates. Two researchers (AMU and QAK) did the manual data extraction and independently performed screening on the articles’ titles and abstracts. We included English-language economic studies of COVID-19 vaccines in countries with COVID-19 vaccination programmes, corresponding to the PICOS eligibility criteria (population – countries providing COVID-19 vaccination, intervention – COVID-19 vaccination, comparison – none, outcome – cost-effectiveness ratio, and study design – full economic evaluation studies, i.e. cost minimization analysis, cost-benefit analysis (CBA), cost-effectiveness analysis (CEA), and cost-utility analysis (CUA)). We excluded review articles, case reports, conference proceedings, non-peer-reviewed papers, opinion pieces, letters to the editor, and commentaries. AMU and QAK retrieved and reviewed the full texts of potentially eligible articles. FR and DN double-checked the results of the study selection. Any disagreements were resolved by discussions with another reviewer (NZ). **Figure 1** shows the PRISMA flow diagram for the study selection process.

### Data collection and quality assessments

The data from the included studies were manually extracted in Microsoft Excel (Microsoft Inc, Seattle WA, USA) using a predetermined format. From each included study, information regarding characteristics of the studies, i.e. information on authors, year of publication, title, country, study objectives, type of study, data collection, and outcome measure, including incremental cost-effectiveness ratio (ICER), quality-adjusted life years (QALYs), disability-adjusted life years (DALYs), life years gained (LYG), and other intermediate measures, were extracted. Moreover, we also documented methodological characteristics, i.e. study perspectives, intervention, and comparator, time horizon, discount rate, choice of model, and sensitivity parameters. Vaccine information comprising vaccination strategy, duration of vaccine protection, vaccination coverage, and vaccine effectiveness was also obtained. In addition, the following cost elements and primary results from each study were documented. All costs were converted to reflect 2022 US$ using the Campbell and Cochrane Economics Methods Group-the Evidence for Policy and Practice Information (https://eppi.ioe.ac.uk/costconversion/default.aspx) Centre Cost Converter Software.

The reporting quality of each included study was assessed using the recent version of the Consolidated Health Economic Evaluation Reporting Standards (CHEERS) 2022 statement [19]. The checklist comprised 24 items classified into six categories, namely, title and abstract, introduction, methods, results, discussion, and others. We calculated a percentage score with the underlying assumption that all criteria were weighted equally after excluding the criteria that were not applicable. Studies were assigned 1 point for reporting the item, 0.5 for partially reporting, and 0 for not reporting. Studies were categorized as “high quality” if they met at least 75% of these standards, “moderate” if they met between 50% and 75% of relevant standards, and “low” if less than 50% [20]. Since this assessment only measures the quality of reporting, the fact of unreported items does not imply poor study quality. This process involved a discussion by all researchers to ensure the accuracy of the findings.

## RESULTS

### Study selection

Through the database search, we retrieved 203 records from MEDLINE/PubMed and 160 records from EBSCO. After eliminating 67 duplicates, we screened 296 records and selected 31 for full-text screening. We further excluded six articles because they were not economic evaluation studies and did not explicitly involve vaccine efficacy. Consequently, we identified 25 articles for the final review (**Figure 1**).

### Study characteristics

**Table 1** provides the characteristics of the 25 included studies, 23 of which were single-country studies, 11 were from LMICs [21-25,29,35,38-40,43,44], and 12 were studies from high-income countries (HICs) [26-28,30-35,37,41,45]. The two remaining studies were conducted in multiple countries; the first one comprised four analyses from LMICs and two analyses from HICs [36], while the other study comprised 12 analyses in LMICs [42]. Sixteen studies were conducted in 2021 [21-28,30-33,35-37,41], while nine studies were conducted in 2022 [29,34,38-40,42-45]. Most of the included studies (21/25) aimed to estimating the economic evaluation of different vaccination strategies [21-30,32,33,35-38,40,42-45], while two studies were conducted with the goal of estimating the economic evaluation of vaccination vs no vaccination [34,41]. Only one study assessed the economic impact of booster vaccination [31], while another compared the intradermal vaccine with the intramuscular vaccine [39].

**Table 1:** General characteristics of included studies

Author, year	Country	Model type	Type of study	Vaccination coverage	Time horizon	Outcome measure	Sensitivity analysis
Reddy et al., 2021 [21]	South Africa	Dynamic state-transition Monte Carlo microsimulation model	CEA	At least 40%	360 d	YLS	One-way and PSA
Hagens et al., 2021 [22]	Turkey	Age-structured deterministic dynamic compartmental model	CUA	70%	1 y	QALYs	One-way
Pearson et al., 2021 [23]	Pakistan	Compartmental transmission model	CUA	NR	10	DALYs	One-way
Vaezi & Meysamie, 2021 [24]	Iran	Epidemiological model	CUA	NR	2-3 mo	DALYs	NR
Suphanchaimat et al., 2021 [25]	Thailand	Deterministic system dynamics and compartmental models	CEA	24%-29% for low risk and 100% for high risk and special population	365 d	Case averted, death averted	One-way
Sandmann et al., 2021 [26]	United Kingdom	Age-structured dynamic transmission and economic model	CBA	75%	10 y	Net monetary value	PSA
Kirwin et al., 2021 [27]	Canada	Epidemiological model	CBA	40%	4 mo	Net monetary value	NR
López et al., 2021 [28]	Catalonia	Epidemiological model	CBA	NR	9 mo	Net monetary value	NR
Fernandes et al., 2022 [29]	Brazil	Markov model	CUA	NR	289 d	QALYs	PSA
Debrabant et al., 2021 [30]	Denmark	Dynamic transition model using a SEIR (susceptible, exposed, infectious, recovered) structure	CUA	15%, 25%, and 40%	6 mo	QALYs	One-way
Padula et al., 2021 [31]	US	Markov model	CUA and BIA	NR	1 y	QALYs	PSA
Bartcsh et al., 2021 [32]	US	Computational model (transmission and age- stratified clinical and economics outcome model)	CUA	30%-50%; 50%-70%; and 70%-90%	180 d, 270 d, and 360 d	QALYs	One-Way
Kohli et al., 2021 [33]	US	Markov model	CUA	34.9% for 18-49 y old; 47.3% for 50-64 y old; and 68.1% for >65 y old	1 y	QALYs	DSA
Li et al., 2022[34]	US	Markov model	CUA	67.37%	6 mo	QALYs	PSA
Wang et al., 2021 [35]	Taiwan	Markov model	CUA, CBA	70%	180 d	QALDs, benefit-cost ratio	One-way
Jiang et al., 2021 [36]	Hongkong Indonesia China Philippines Singapore Thailand	Decision tree	CUA	70%	1 y	QALYs	One-way and PSA
Marco-Franco et al., 2021 [37]	Spain	Mathematical Modeling (Best Adjustment of Related Values (BARV) method)	CUA	70%	5 y	QALYs	One-way
Morales-Zamora et al., 2022[38]	Colombia	Markov discrete time	CUA	NR	1 y	DALYs	DSA
Mungmunpuntipantip and Wiwanitkit, 2022[39]	Thailand	NR	Cost-Analysis and CEA	NR	NR	SARS-CoV-2 Anti-RBD antibody response	NR
Orangi et al., 2022 [40]	Kenya	Age-structured transmission model	CUA	30%	1.5 y	DALYs	One-way
Orlewska et al., 2021 [41]	Poland	Markov model	CUA	100%	1 y	QALYs	DSA
Siedner et al., 2022 [42]	Bangladesh, Republic of Congo, Egypt, Ethiopia, Indonesia, Kenya, Myanmar, Nigeria, Pakistan, Philippines, Tanzania, Vietnam	CEACOV model	CEA	15%, 30%, 45%, and 60%	360 d	YLS	One-way
Siquera et al., 2022 [43]	Brazil	Probabilistic epidemiological model	CBA	NR	6 mo	Cost-benefit ratio	NR
Wang et al., 2022 [44]	Thailand	Age-structured transmission dynamic model	CUA	60%	1 y	QALYs	One-way and PSA
Xiong et al., 2022 [45]	Hongkong	Markov model	CUA	70%	1 y	QALYs	One-way and PSA

BIA – budget impact of analysis, CBA – cost-benefit analysis, CEA – cost-effectiveness analysis, CUA – cost-utility analysis, DALYs – disability-adjusted life years, DSA – deterministic sensitivity analysis, ICER – incremental cost-effectiveness ratio, NR – not reported, PSA – probabilistic sensitivity analysis, QALYs – quality-adjusted life years, QALD – quality-adjusted life days, YLS – years of life saved, y – years, mo – months

### Methodological characteristics

CUA was conducted in 15 studies, 11 of which used QALYs as an outcome [22,29,30,32-34,36,37,41,44,45], while four used DALYs [23,24,38,40]. Four studies conducted CBA, with most using net monetary value as the outcome measure [26-28], while only one study used cost-benefit ratio as the outcome measure [43]. For studies using CEA, two articles used year of life saved (YLS) as the outcome [21,42] while another used averted cases and deaths [25]. One study conducted CUA using QALYs as the outcome alongside budget impact analysis (BIA) [31]. SARS-CoV-2 anti-RBD antibody response was chosen as the outcome in a study using CEA, which also conducted a cost analysis [39]. One remaining study used two concurrent economic analyses – CEA with quality-adjusted life days (QALD) as outcomes and CBA with cost-benefit ratios [35] (**Table 1**).

Ten studies applied the dynamic transmission model [21-23,25,26,30,32,40,42,44], eight applied the Markov model [29,31,33-35,38,41,45], and four studied the epidemiological model [24,27,28,43] for modeling the evaluation. One study utilized a decision tree [36] and another used simplified mathematical modeling [37]. One study did not report the type of modeling used [39]. The short time horizon was reported in most studies, i.e. two, three, four, six, and nine months [24,27-30,32,34,35,43] and one year [21,22,25,31,33,36,38,40-42,44,45], although a longer time horizon was also reported in three studies [23,26,37]. However, one study did not report a time horizon [39]. For time horizons of more than one year, a discount rate must be mentioned [46]. More than half of the studies (13/25) did not report the discount rate for costs and effects [21,24,25,28,29,31,35,38,39,43-45]. Most studies set similar discount rates for costs and effects at 3.5% [37], 3% [22,23,32-34,36,40], 1.5% [27], 3%-5%[26], and 2%-4%[30]. While two studies reported 3% [42] and 3.5% [41] discount rates only for the effects.

Regarding perspectives, 12 studies used a healthcare perspective [25,27,29-31,33,34,36,38,41,45,47], four used a societal perspective [36,37,40,44], two used a policymaker perspective [21,24], and one adopted the payer perspective [42]. Additionally, the remaining studies used more than one perspective, i.e. healthcare and societal perspectives or societal and payer perspectives [22,23,28,32,35]. Only one study did not report the perspective adopted [39] (**Table 2**). At least four studies specified a vaccination coverage of less than 50% [21,27,30,40], eight had a vaccination coverage of 50%-75% [22,26,34-37,44,45], and one specified a vaccination coverage of 100% [41]. Moreover, three studies presented a range of two to three categories of vaccination coverage each [25,32,42]. Eight studies, however, did not report on vaccination coverage [23,24,28,29,31,38,39,43].

**Table 2:** Cost elements and main findings of included studies

Author	Perspective	Discount rate		Cost data				Willingness to pay threshold	Primary result	Parameter in sensitivity analysis
		**Cost**	**Outcome**	**Direct cost**			**Indirect cost**			
				**Medical cost**		**Non-medical**				
Reddy et al., 2021 [21]	Healthcare (public and private)	NA	NA	Vaccination cost, hospital and ICU cost		NR	NR	One GDP per capita or published opportunity cost	COVID-19 vaccination program would reduce infections and deaths, and likely reduce overall healthcare costs (in ICERs of US$520/YLS) in South Africa across a range of possible scenarios, even with conservative assumptions around vaccine effectiveness.	One-way: prior immunity, reproduction number, cost per person vaccinated; multi-way: vaccination pace
Hagens et al., 2021 [22]	Healthcare and societal	NA	3%	Healthcare costs of hospitalisation, the ICU stay, and pharmacotherapy at home and vaccination		NR	Productivity losses due to sickness leave and premature death	One GDP per capita	Vaccination is cost-effective if the vaccine’s efficiency in preventing transmission is equal to or less than 50% of its effectiveness in preventing transmission with an ICER US$511/QALYs and US$1045/QALYs.	Total susceptible persons and vaccination cost
Pearson et al., 2021 [23]	Health system (healthcare and partial societal)	3%	3%	Vaccine procurement price per dose, syringes and safety boxes, cold chain costs per dose,		Wastage, freight, human resources per dose, transport per dose, social mobilization per dose	NR	NR	At 1 y distribution, US$3/dose vaccine yielded 70% efficacy and 2.5-y duration of protection is likely to avert around 0.9 (95% CrI = 0.9, 1.0) million cases, 10.1 (95% CrI = 10.1, 10.3) thousand deaths, and 70.1 (95% CrI = 69.9, 70.6) thousand DALYs, with an ICER of US$27.9 per DALYs. Covid 19 vaccination is highly cost-effective and cost-saving in Sindh Province, Pakistan, if the vaccine prices<US$10/dose and the infection occurs at short term (not more than 5 y or lifelong).	Vaccine price
Vaezi and Meysamie, 2021 [24]	Policy maker	NR	NR	Cost of hospitalisation, cost of vaccine per dose		NR	NR	One GDP per capita	The ICER for a vaccination with COVID-19 vaccines was estimated at US$6.2 to US$121.2 to avert one DALYs and US$566.8 to US$10 957.7 per one death. All vaccines are cost-effective except CoronaVac and Janssen.	NR
Suphanchaimat, et al., 2021 [25]	Provider	NA	NA	Treatment unit cost per, vaccination cost, and vaccine administration costs		NR	NR	NR	The migrant-centric vaccination policy scenario received the lowest incremental cost per one case or one death averted compared with the other scenarios. The Thai-centric policy scenario yielded an incremental cost of US$2282 per one life saved, while the migrant-centric policy scenario produced a comparable incremental cost of US$317.4. A migrant-centric policy yielded the smallest volume of cumulative infections and deaths and was the most cost-effective scenario.	Values of the reproduction number
Sandmann et al., 2021 [26]	Healthcare	3%-5%	3%-5%	Hospital admissions cost (ICU and non-ICU), enhanced personal protective equipment cost, visits to general practitioners cost, remote helpline calls cost, adverse events following immunization cost, vaccine administrations cost, and vaccine costs		NR	NR	ICER threshold<US$22 476	Introducing vaccination leads to incremental net monetary values ranging from US$17.6 billion to US$4899 billion in the best-case scenario and from -US$1.61 billion to US$83.4 billion in the worst-case scenario	Vaccination vs no Vaccination
Kirwin et al., 2021 [27]	Healthcare	1.50%	1.50%	Vaccination cost		NR	NR	NR	Using prioritisation of those over the age of 60 y at high risk of poor outcomes, active cases are reduced by 17% and net monetary benefit dollars, relative to no vaccine increased by US$263 million dollars, relative to no vaccine	NA
López et al., 2021 [28]	Societal and healthcare	NA	NA	Vaccination cost		Vaccination campaign cost	NR	NR	The benefit/cost ratio is estimated at 3.4 from a social perspective and 1.4 from a health system perspective. The social benefits of vaccination are estimated at US$152.99 per vaccine dose (US$26.14 from the perspective of the health system).	NA
Fernandes et al., 2022 [29]	Public health system	NA	NA	Medical visits, diagnostic tests, hospital stay (ward		NR	NR	US$3436.38	The vaccines showed incremental cost-utility ratios ranging from US$4525.81/QALYs (Oxford) to US$3469.79/QALYs (CoronaVac) and considered cost-effective.	Vaccine efficacy
Debrabant et al., 2021 [30]	Healthcare sector's perspective	2%-4%	2%-4%	ICU cost hemodialysis, laboratory tests, imaging tests	NR		Productivity loss	NR	Inclusion of the elderly population aged ≥60 y was more cost-effective than a vaccination strategy that targeted a population aged <60 y old only, when productivity losses were not included.	Vaccine efficacy
Padula et al., 2021 [31]	Healthcare sector perspective	NR	NR	Unit cost of each vaccine dose.		NR	Productivity loss	US$100 000/QALYs	Vaccination compared to do nothing has a dominant ICER value with a program cost of $13 042 and a budget impact cost of $40 so that it can be stated that vaccination is cost-effective.	Vaccine cost; vaccination rate; and vaccine efficacy.
Bartcsh et al., 2021 [32]	The third- party payer and societal perspective	3%	3%	Vaccination cost and hospitalisation cost		NR	Productivity losses due to absenteeism resulting from COVID-19 illness	ICER below US$50 000	1. Achieving 50% coverage in 180 d with a 70% efficacious vaccine resulted in a decrease of 20.9 million cases, 775 980 hospitalisations, and 91 660 deaths and a gain of 977 730 QALYs.; 2. Shortening to 180 d (vs 270 d) decreased cases by 2.6 million and deaths by 11 300, saving by $5.3 billion in total costs.	Vaccine efficacy and vaccine's reproduction number
Kohli et al., 2021 [33]	Healthcare	3%	3%	Vaccine cost, vaccine administration cost, COVID-19 treatment cost, and hospitalisation cost		NR	NR	US$50 000 to US$150 000 per QALYs gained	1. The incremental cost per QALYs gained for the US adult population was US$8200 vs no vaccination; 2. For the tiers at highest risk of complications from COVID-19, such as those ages 65 y and older, vaccination was cost-saving compared to no vaccination; 3. The cost per QALYs gained increased to over $94 000 for those with a low risk of hospitalisation and death following infection.	Infection incidence, vaccine price, the cost of treating COVID-19, and vaccine efficacy
Li et al., 2022 [34]	Healthcare	3%	3%	Hospital administration cost, vaccines cost, and PCR test cost		NR	NR	ICER bellow US$50 000	The booster strategy is a cost-saving strategy. The strategy would prevent 3.8 COVID-19 deaths, indicating a requirement of US$904 382 to prevent 1 COVID-19 death.	Population incidence of COVID-19 at one time
Wang et al., 2021 [35]	Healthcare and societal	NR	NR	Confirmatory diagnosis cost, vaccine price per dose, vaccine administration per dose, hospitalisation cost per day, vaccine jab cost per half-day			Adverse effect due to vaccination	ICER below US$50 000	1. Cost-utility analysis result: Moderna = -US$321.14/QALD, Pfizer = -US$356.75/QALD, AstraZeneca = -US$341.44.;2. Cost-benefit analysis result: Moderna = US$13, Pfizer = US$23, and AstraZeneca = US$28.	Hospitalisation fee and proportion of asymptomatic cases
Jiang et al., 2021 [36]	Societal	3%	3%	Vaccination cost and medical cost		NR	Productivity loss	One time GDP per capita	Population immunization programs using inactivated COVID-19 vaccines may be not only cost-effective but also cost- saving in Hong Kong SAR, Indonesia, mainland China, Philippines, Singapore, and Thailand with US$105.18, US$98.15, US$99.70, US$60.48, US$112.00, and US$103.47 QALYs compared with no vaccination in Hong Kong SAR, Indonesia, mainland China, Philippines, Singapore, and Thailand, respectively.	Vaccine efficacy against COVID-19 cases by severity
Marco-Franco et al., 2021 [37]	Buyer	3.50%	3.50%	Hospital administration, hospitalisation, vaccine cost		Transportation cost to hospital	NR	One to three times GDP per capita	Vaccination of about 70% of the Spanish population, with a conservative 70% ratio of efficacy and two shots, will result in US$5042.42 per QALYs gained.	Discount rate and mortality of COVID-19
Morales-Zamora et al., 2022 [38]	Healthcare system	NR	NR	Treatment of the patients and vaccine acquisition		NR	NR	One time GDP per capita	Prioritization of high-risk population reduces symptomatic cases by 3,4% and deaths by 20,1% compared with no vaccination with ICER value is US$3339 per DALYs	Probability of having symptom for age 70+ and yearly immunity loss
Mungmunpuntipantip and Wiwanitkit, 2022 [39]	NR	NR	NR	Vaccine administration		NR	NR	NR	Cost-utility and cost-safety analysis also show that the cost per utility and cost per safety values for intradermal vaccination are lower than those of intramuscular vaccination with utility value 0.207 and safety value 9.67	NR
Orangi et al., 2022 [40]	Societal	3%	3%	Vaccination cost, treatment cost		Freight, wastage, transport	Productivity loss due to illness and mortality	US$919.11	Slow roll-out at 30% coverage largely targets those over 50 y and resulted in 54% fewer deaths (8132 (7914 to 8373)) than no vaccination and was cost saving (incremental cost-effectiveness ratio, ICER = -US$1343 (-US$1345 to -US$1341) per DALYs averted). Rapid roll-out with 30% coverage averted 63% more deaths and was more cost-saving (ICER = -US$1607 (-US$1609 to -US$1604) per DALYS averted) compared with slow roll-out at the same coverage level	Vaccine procurement (in 50% coverage, both rapid and non-rapid vaccination pace)
Orlewska et al., 2021 [41]	Public healthcare	NR	3.50%	Vaccine cost, vaccine administration cost, COVID-19 treatment cost, and hospitalisation cost		NR	NR	Three times GDP per capita	In the base case analysis, the incremental cost per QALYs gained associated with vaccinating the whole population is US$3688.71. For individuals aged 60-69 y and >80 y vaccination is less costly and more effective than no vaccination. The incremental cost per QALYs gained when vaccinating individuals aged 40-49 and 30-39 y is US$16 517 and US$39 500, respectively. In the general population and in younger subpopulations the incremental cost-effectiveness ratio is most sensitive to the vaccine effectiveness, vaccine price, and SARS-CoV-2 infection rates.	Vaccine effectiveness, vaccine price, and SARS-CoV-2 infection rates (in general population and younger subpopulation)
Siedner et al., 2022 [42]	Donor	0%	3%	Vaccine cost		Program cost	NR	There is no threshold but ICER could be in the range US$670/YLS for achieving at least 15% coverage to US$7820/YLS for 16 achieving at least 60% coverage in an omicron-like scenario	In the omicron-like scenario, increasing current vaccination coverage to achieve at least 15% in 13 each of the 91 LMICs would prevent 11 million new infections and 120 000 deaths, at a cost of 14 US$0.95 billion, for an incremental cost-effectiveness ratio (ICER) of US$670/y-of-life saved 15 (YLS). Increases in vaccination coverage to 60% would additionally prevent up to 68 million 16 infections and 160 000 deaths, with ICERs<US$8000/YLS	Vaccination program costs
Siquera et al., 2022 [43]	Public health authorities	NR	NR	Cost of vaccination		Acquisition herd immunity cost, cost of number of death	NR	Achieve 70% herd immunity	AstraZeneca has the best cost-benefit when prioritizing acquisition costs, while Pfizer is the most cost-beneficial when prioritizing the number of deaths.	NR
Wang et al., 2022 [44]	Societal perspectives	NR	NR	Medical cost, cost of vaccination, cost of vaccine Administration, cost of vaccine acquisition cost, cost of COVID-19 screening, cost of vaccine-related adverse event		Cost of vaccine supply chain, cost of mask, cost of hand sanytizer, cost of contact tracing, cost of quarantine, cost of social distancing,	NR	Below US$17 499	1. COVID-19 vaccines that block infection combined with social distancing were cost-saving regardless of the target population compared to social distancing alone (with no vaccination); 2. COVID-19 vaccines that reduces severity (including hospitalisation and mortality) were cost-effective when the elderly were vaccinated, while vaccinating the high incidence group was not cost-effective with this vaccine type; 3. Regardless of vaccine type, higher vaccination coverage, higher efficacy, and longer protection duration were always preferred.	Vaccine efficacy
Xiong et al., 2022 [45]	Healthcare sector perspective	NR	NR	Polymerase chain reaction tests, hospitalisation care, and ICU care		NR	Productivity loss	Below US$240 963	1. The ICER of the vaccination program before Omicron period was found to have a cost of US$5 383 060 per QALYs (not cost-effective in the context before the Omicron wave); 2. The ICER of the vaccination program in Omicron period was US$74 721 (cost-effective in the context of the Omicron)	Vaccination rate

NA – not available, NR – not reported, QALYs – quality-adjusted life years, QALD – quality-adjusted life days, ICER – incremental cost-effectivens ratio, YLS – years of life saved, DALYs – disability-adjusted life years, QALD – quality-adjusted life days, ICU – intensive care unit, CrI – credible interval, GDP – gross domestic product, y – year, mo – months

Regarding the possibilities of uncertainty, five studies did not conduct sensitivity analyses [24,27,28,39,43]. Those that did commonly used deterministic sensitivity analyses, particularly one-way sensitivity analyses [22,23,25,30,32,33,35,37,38,40-42]. Four studies performed probabilistic sensitivity analysis (PSA) [26,29,31,34]. The remaining included studies used more than one sensitivity analysis, i.e. PSA and one-way sensitivity analysis [21,36,44,45].

### Cost estimation

Regarding cost components, the direct medical costs were mostly vaccination costs and hospitalisation or ICU treatment costs related to COVID-19 infection [21-24,26-28,32-38,40,42]. Three studies considered diagnostic testing costs in direct medical costs [29-31]. Only eight studies reported direct non-medical costs, including vaccine wastage, freight, human resources, transportation, social mobilization, contact tracing, quarantine, social distancing, and vaccination campaigns [23,28,35,37,40,42-44]. The indirect costs considered were those associated with economic productivity loss because of COVID-19 [22,30-32,36,40,45]. **Table 2** summarizes the detailed information about the cost elements of the included studies.

### Primary results

The value of the willingness-to-pay (WTP) threshold differed depending on the study. Several studies used their own thresholds [26,33-35,40,42,43,45,48-50]; some referred to one to three times the gross domestic product (GDP) per capita as the threshold value [21,22,24,36-38,41] while others did not define it [23,25,28,30,39,41] Overall, all studies suggested that vaccination to prevent the COVID-19 pandemic was a cost-effective strategy. Each analysis used a different evaluation to determine whether the use of vaccinations was considered cost-effective, e.g. by considering the procurement of the vaccine program compared with the absence of a vaccination program [21,26,33,36,39,41,45,48,50], the coverage of the vaccine used [37,40,42,49], the existence of priority vaccines for specific populations [25,30,33,34,38,41], the efficacy of many vaccines on the market [23,24,29,35,41,43], and the provision of boosters following vaccination [34].

Several studies did not conduct sensitivity analysis to determine the uncertainty of the analysis [24,28,39,41,43], but most did. Reproduction number [21,25,49], vaccine price/cost [22,23,33,41,48], vaccination program [26,42,45], vaccine efficacy [29,30,33,36,40,41,48-50], population/infection incidence [33,34,38], hospitalisation fee [35], and discount rate and mortality [37] were the most reported sensitive parameters in the sensitivity analysis. Vaccine cost was one of the most essential factors in determining cost-effectiveness. All the included studies reported vaccine prices, and most of them calculated the average price to obtain the effectiveness of vaccination. Additionally, the vaccine administration cost was also considered, varying from US$0.50 to US$20.16 [25,30,33-36,47]. According to the CHEERS 2022 checklist, 14 studies were classified as high quality [21-23,25-28,30,31,36,38,40,42]_,_ while 10 were classified as moderate quality [24,29,32,34,35,37,41,43-45]. Only one study was categorized as low in quality of reporting [39]. The abstract and results sections were almost entirely reported in all studies. Most studies have discussed vaccination perspectives and time horizons, but justifications have rarely been mentioned. All studies provided the currency used, but the years of costing and conversion were not fully reported. Regarding heterogeneity, only a few studies described techniques for estimating how the study's results differ for subgroups. The role of the study's funder was also underreported, even though almost all studies reported the source of funding.

## DISCUSSION

We identified 25 studies on the economic evaluation studies of COVID-19 vaccination globally. The results showed that the vaccination programs would be cost-effective and even cost-saving compared to no vaccination at all, even when the efficacy of vaccines largely varied, was assumed to be relatively low, and when only a specific age cohort was targeted. Moreover, vaccine effectiveness, costs, and coverage were among the most influential parameters for estimating the cost-effectiveness. We also found variability regarding input parameters in all included studies, eg, the choice of modeling, perspectives, cost components, vaccine coverage, target population, and discount rate.

Vaccination is considered the most cost-effective intervention to fight the COVID-19 pandemic. Most economic evaluation studies on COVID-19 vaccination were from HICs and middle-income countries (MICs), while studies in low-income countries (LICs) were very limited. Most evaluation studies used a decision analytic modeling approach to predict the cost-effectiveness of COVID-19 vaccination. Approximately half of the studies using modeling used dynamic models in the evaluation, which may consider herd immunity and dynamic shifts in the age distribution of the population, thus providing a representation of infectious disease transmission. The models’ assumptions and parameters relating to the direct and indirect costs and vaccine efficacy varied. COVID-19-related costs were determined by the perspectives used in the studies. Societal perspectives could give more comprehensive data in the decision-making process because both direct and indirect costs, such as productivity loss, were considered in the analysis. In contrast, the healthcare perspective only considers direct costs. However, as the data were limited during the COVID-19 pandemic, the healthcare perspective was mostly used, as indicated in most included studies. Most studies used a one-year time horizon or less, considering the viral infection’s nature and the expected effects of vaccination. Vaccination is supposed to be more cost-effective in a shorter time horizon than other interventions such as social distancing [51]. A study that used a short time horizon (≤1 year) did not have to consider a discount rate in the analysis [46]. A longer time horizon was used to determine a longer effect, as done, for example, in the study by Pearson et al. [23], which considered campaign duration and duration of natural immunity for 10 years. Thus, discounting the costs and effects became necessary [46].

Most studies reported vaccine efficacy to be around 60%-95%. These findings are correlated with the fact that vaccination could decrease hospitalisation rate, disease severity, and mortality [13,52]. A previous study also reported that a COVID-19 vaccination could minimize adverse outcomes [52]. Although vaccination could prevent coronavirus transmission, not all countries can afford the same type of vaccines (different types may result in different efficacy) and the number of rounds or doses to be administered. Thus, the economic evaluation becomes important for policymakers to decide on the implementation strategies. Our results indicated that COVID-19 vaccination can be considered a cost-effective or cost-saving intervention, even in LMICs. Combined with lockdown and physical distancing, vaccination is estimated to have decreased 148 million cases and 3.1 million deaths [26]. Vaccines can reduce community transmission without doing physical distancing in the future [26]. The analysis also summarized that mass vaccination campaigns against COVID-19 are cost-saving [28]. From an economic perspective, vaccination campaigns have high social returns [28]. Regarding benefits, the speeding up of vaccination coverage could decrease the number of cases and deaths [32].

Many aspects can influence the priority of conducting COVID-19 vaccination during the beginning of pandemic eg, prioritization criteria, vaccine effectiveness and coverage, and implemented policies [27]. For instance, in Thailand, prioritizing persons at risk of contracting COVID-19 exhibited a more cost-effective effect [25]. In the USA, if the analysis did not consider the productivity loss, prioritizing vaccines for people older than 60 years was more cost-effective [33]. However, the analysis in Denmark suggested that even when the loss of productivity is considered, the scenario to prioritize vaccination for people younger than 60 years can still be considered cost-effective [30].

Because of limited resources, cost is an essential aspect of estimation in any economic evaluation study. Costs are calculated to estimate resource scarcity, which occurs when resources used for one purpose are no longer available for use in another. Decision-makers must choose appropriate WTP thresholds in economic evaluations. Making accurate estimations in WTP would assist policymakers in making informed decisions regarding the healthcare allocation [53]. If a specific WTP is not available, a threshold of less than three times the country’s annual GDP per capita is recommended by the World Health Organization’s Choosing Interventions that are Cost-effective (WHO-CHOICE) project, with interventions that cost less than one time the country’s annual GDP per capita being considered highly cost-effective [54]. The WTP research is predicated on the notion that societal preferences should be considered when making choices on how to distribute resources in the health system [55].

Previous studies also suggested that vaccination against COVID-19 was more cost-effective than no vaccination at all. The COVID-19 vaccine is superior in LMICs in terms of clinical effectiveness and economic value. Vaccination programs have shown to be the most cost-effective strategy to stop the COVID-19 pandemic under any circumstances or situation. The efficacy of the vaccine, the priority of administration in a certain group, and vaccination coverage are the three primary factors for deciding whether a vaccine is cost-effective or not. Herd immunity, which lowers mortality in COVID-19 patients, is influenced by vaccine efficacy and vaccination coverage, whereas population priorities have an effect, since vaccines might be useless if not administered to the correct populations [22-25,29,30,33,35,38,40,41,43,49].

Knowing the most influential parameter in sensitivity analyses enables us to identify the factors that have a significant effect on the ICER values. If the results of the sensitivity analyses are consistent with the base-case analysis’ results and lead to similar conclusions on the cost-effectiveness of various strategies, one can be confident that any uncertainty about the model input has been minimized [56].

Despite its limitation, such as the included studies’ heterogeneity, policymakers could use our study to review evidence from all published research on economic evaluations of COVID-19 vaccination to conduct vaccination programs or to use it as a basis for a possible future pandemic. Nonetheless, COVID-19 vaccination has been deemed a cost-effective intervention in many countries. Our findings suggest that optimal vaccine allocation will be determined by current public health policies and their effects on a given population.

## CONCLUSIONS

COVID-19 vaccination is considered one of the most cost-effective interventions to fight COVID-19 globally. Most studies reported the values of vaccine efficacy to be 65%-75%. The results of economic evaluation in the included studies indicate that COVID-19 vaccination could be considered a cost-effective or cost-saving intervention, even in LMICs. Given the disparities in affordability across countries, prioritization has become crucial to consider. Our study provides insights for conducting effective vaccination that will be helpful to policymakers, particularly as the next possible pandemic approaches.

## Additional material


Online Supplementary Document
